# Genome-wide identification of key modulators of gene-gene interaction networks in breast cancer

**DOI:** 10.1186/s12864-017-4028-4

**Published:** 2017-10-03

**Authors:** Yu-Chiao Chiu, Li-Ju Wang, Tzu-Hung Hsiao, Eric Y. Chuang, Yidong Chen

**Affiliations:** 1 Greehey Children’s Cancer Research Institute, University of Texas Health Science Center at San Antonio, San Antonio, TX 78229 USA; 2 Graduate Institute of Biomedical Electronics and Bioinformatics, National Taiwan University, Taipei, Taiwan; 3 Research Center for Chinese Herbal Medicine, China Medical University, Taichung, Taiwan; 4 Department of Medical Research, Taichung Veterans General Hospital, Taichung, Taiwan; 5 Bioinformatics and Biostatistics Core, Center of Genomic Medicine, National Taiwan University, Taipei, Taiwan; 6 Department of Epidemiology and Biostatistics, University of Texas Health Science Center at San Antonio, San Antonio, TX USA

**Keywords:** Modulated gene interactions, Modulator genes, Gene interaction networks, Genome-wide analysis, Breast cancer

## Abstract

**Background:**

With the advances in high-throughput gene profiling technologies, a large volume of gene interaction maps has been constructed. A higher-level layer of gene-gene interaction, namely modulate gene interaction, is composed of gene pairs of which interaction strengths are modulated by (i.e., dependent on) the expression level of a key modulator gene. Systematic investigations into the modulation by estrogen receptor (ER), the best-known modulator gene, have revealed the functional and prognostic significance in breast cancer. However, a genome-wide identification of key modulator genes that may further unveil the landscape of modulated gene interaction is still lacking.

**Results:**

We proposed a systematic workflow to screen for key modulators based on genome-wide gene expression profiles. We designed four modularity parameters to measure the ability of a putative modulator to perturb gene interaction networks. Applying the method to a dataset of 286 breast tumors, we comprehensively characterized the modularity parameters and identified a total of 973 key modulator genes. The modularity of these modulators was verified in three independent breast cancer datasets. *ESR1*, the encoding gene of ER, appeared in the list, and abundant novel modulators were illuminated. For instance, a prognostic predictor of breast cancer, *SFRP1*, was found the second modulator. Functional annotation analysis of the 973 modulators revealed involvements in ER-related cellular processes as well as immune- and tumor-associated functions.

**Conclusions:**

Here we present, as far as we know, the first comprehensive analysis of key modulator genes on a genome-wide scale. The validity of filtering parameters as well as the conservativity of modulators among cohorts were corroborated. Our data bring new insights into the modulated layer of gene-gene interaction and provide candidates for further biological investigations.

## Background

As technologies of high-throughput profiling advance, a large volume of post-transcriptional gene interaction maps has been established. For instance, the Kyoto Encyclopedia of Genes and Genomes (KEGG) is a knowledge-based curation of abundant genomic pathways among species [1]. Such maps provide better understanding to the molecular signaling in cells, however, they are typically derived under a certain cellular condition in a single model cell line. In light of the dynamicity and complexity of gene interactions (reviewed in [2, 3]), a higher-order layer of interaction networks that considers gene-gene relationships modulated by (i.e., dependent on) key modulator genes, namely modulated gene interaction, was proposed (reviewed in [4]). In this sense, interaction of two genes can be strengthened specifically when a modulator gene is expressed at high or low abundance. The scenario provides flexibility and interpretability to condition-specific and dynamic interaction networks.

In breast cancer, estrogen receptor (ER) is the best-studied modulator gene. It governs the coexpression among several keratin genes in breast cancer patients [5]. Also, topological and temporal changes were observed in a transcription factor interaction network of MCF7 cells upon 17β-estradiol stimulation [6]. A comprehensive in silico investigation revealed compact gene-gene and function-function interaction networks modulated by ER and discovered the prognostic value of ER-modulated interaction between TGFβ and NFκB [7]. By a co-modulation analysis, we previously showed ten experimentally chosen genes jointly modulated up to two-thirds of all gene pairs, with an implication in cellular processes associated with hormone stimulus [8]. Taken together, these reports demonstrate the existence and functional significance of modulated gene interaction, and motivate a comprehensive search for key modulator genes. Based on mutual information, a modulator inference by network dynamics (MINDy) was developed to systematically identify modulators of transcription factor (TF)-target gene interactions [9]. However, due to a heavy computational burden caused by permutation-based assessment of statistical significance, the method was limited to the investigation of specific TFs and relied on prior knowledge of TF-target relationships. Recently, we exploited the transformability of Pearson correlation coefficients to devise a highly efficient modulated gene/gene set interaction (MAGIC) analysis and realized the exploration into genome-wide interaction networks modulated by a modulator gene [7]. However, a reverse-engineering study for a genome-wide identification of key modulators is still lacking.

In the present study we proposed a systematic workflow that incorporates the MAGIC algorithm to analyze gene expression profiles of breast tumors. Comparing samples with high and low expression levels of a modulator, four modularity parameters were designed to measure modulator-dependent changes in gene interaction at two layers. One was focused on the summary of genome-wide changes, while the other assessed the scale and information flow in the core subset of modulated interaction pairs. Genes with significantly high values of parameters were defined as key modulators and validated by three independent cohorts. Functional annotation analysis was performed to study the functional involvement of these modulators. Collectively, this report describes a novel genome-wide search for key modulators in breast cancer and unveils the functional landscape of modulated gene interactions.

## Methods

### Microarray datasets

We downloaded and reanalyzed four public gene expression microarray datasets of breast cancer patients from the Gene Expression Omnibus database [10] and The Cancer Genome Atlas (TCGA). A dataset of 286 lymph-node negative breast tumors (GSE2034) [11], profiled by Affymetrix Human Genome U133A Arrays, was analyzed for the identification of key modulator genes. We validated the findings in three large independent cohorts, GSE2990 [12], GSE4922 [13], and TCGA [14, 15]. Gene-level intensity values of GSE2034, GSE2990, and GSE4922 were calculated by reprocessing of Affymetrix CEL files by Robust Microarray Analysis (RMA) algorithm, representation of each gene by the most informative probe (measured by the coefficient of variation (CV)), and removing non-informative genes, as previously described [7, 8]. For the TCGA dataset, we used pre-normalized level-3 (gene-level) data.

### Model overview

**Fig. 1:**
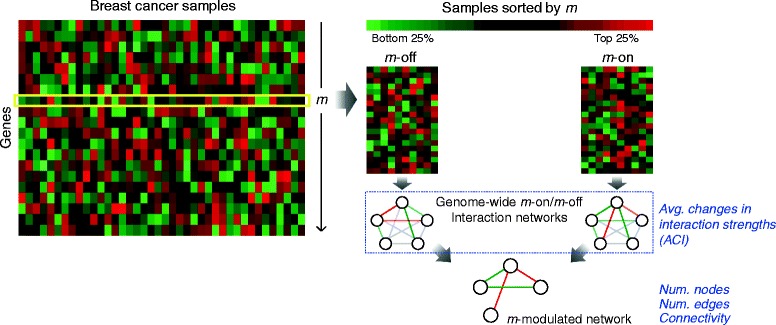
Illustration of a genome-wide identification of modulator genes. In the present study we proposed a workflow to systematically identify key modulators from gene expression profiles. Briefly, for each putative modulator gene *m*, samples are sorted by its expression levels and the top/bottom 25% are defined as *m*-on/off samples. We designed four parameters to measure the modularity of *m*. The ACI score (parameter 1) measures the average change in normalized correlation coefficients between genome-wide gene interaction networks constructed in *m*-on and *m*-off samples. Focusing on the core subset of gene interactions, a *m*-modulated interaction network is built of significantly differentially correlated gene pairs called by MAGIC between the conditions. Three parameters, namely number of nodes, number of edges, and connectivity (i.e., average node degree), are employed to measure the scale and information flow of the core network. The procedures are performed iteratively to analyze each gene in the expression dataset

We devised a systematic workflow for identifying key modulator genes on a genome-wide scale (illustrated in Fig. 1). Four modularity parameters were designed to measure the ability of a gene as a modulator of gene interaction networks. Conceptually, one of the parameters was designed to test whether genome-wide interaction networks formed in samples with high/low expression of a putative modulator gene show an overall change in interaction strengths. The other three parameters measure the size and information flow of the core modulated gene interaction network constructed by core gene pairs of which interaction strengths significantly change between the two groups of samples.

### Analysis of overall changes in genome-wide gene interactions networks

Suppose a gene expression dataset contains expression profiles of *G* genes of *N* samples,

1 $$ \boldsymbol{E}={\left\{{e}_{g,n}\right\}}_{G\times N}, $$

where *e*_*g* , *n*_ denotes the normalized expression level of gene *g* in sample *n*. For a gene *m* (1 ≤ *m* ≤ *G*), we selected two groups of samples for analysis, *m*-on (*M* = 1) and *m*-off (*M* = 0), defined as samples with the highest and lowest 25% of *m*, respectively. In each of the two sample groups, we built genome-wide gene correlation matrices, i.e.,

2 $$ {\boldsymbol{C}}_{\boldsymbol{M}}\left(i,j\right)=\rho \left({\boldsymbol{E}}_{\boldsymbol{M}}\left(i,:\right),{\boldsymbol{E}}_{\boldsymbol{M}}\left(j,:\right)\right), $$

where ***E***_*M*_(*i*, :) and ***E***_*M*_(*i*, :) represent the vectors of expression values of genes *i* and *j*, respectively, for each status of *M*. The matrices were Fisher transformed to the standard normal domain

3 $$ {\boldsymbol{I}}_{\boldsymbol{M}}\left(i,j\right)=\mathcal{F}\left({\boldsymbol{C}}_{\boldsymbol{M}}\left(i,j\right)\right)=\frac{\sqrt{N/4-3}}{2}\mathit{\ln}\left(\frac{1+{\boldsymbol{C}}_{\boldsymbol{M}}\left(i,j\right)}{1-{\boldsymbol{C}}_{\boldsymbol{M}}\left(i,j\right)}\right). $$

Based on the intuition that expressional changes of a key modulator gene can perturb the overall gene interaction, we set the average changes in interaction strengths (*Parameter 1: ACI score*) as the first criterion for key modulator genes:

4 $$ \boldsymbol{ACI}\left(i,j\right)={AVG}_{1<i<G,1\le j<i}\left(\Delta \boldsymbol{I}\left(\boldsymbol{i},\boldsymbol{j}\right)\right), $$

where ∆***I*** = ||***I***_*M* = 1_| − |***I***_*M* = 0_||. Statistical significance of an ACI score was assessed against a null distribution ***D*** ^***ACI***^ by a 10,000-time random permutation of ***E*** with respect to samples:

5 $$ {\boldsymbol{P}}_{\boldsymbol{ACI}}\left(i,j\right)=\frac{\left|\boldsymbol{ACI}\left(i,j\right)>{\boldsymbol{D}}^{\boldsymbol{ACI}}\right|}{10^4}. $$

### Analysis of core modulated gene interactions networks

We also analyzed the core subset of gene interactions modulated by *m* to measure its modularity. Specifically, a *m*-modulated gene interaction network was constructed by the MAGIC method [7]. The method adopts a conjugate Fisher transformation – inverse Fisher transformation scheme to identify gene interaction pairs of which interaction strengths change considerably between *m*-on and *m*-off samples. Briefly, it tests the significance of ∆***I***(*i*, *j*), by a fully derived statistical model (hereafter referred to as MAGIC *P*-value). To ensure that the change is meaningful in biological context, it sets a threshold on the MAGIC score, defined as the change between two correlation coefficients projected from ***I***(*i*, *j*) to the domain with assigned sample size (e.g., average of two groups) by an inverse Fisher transformation:

6 $$ {\boldsymbol{C}}_{\boldsymbol{M}}^{\boldsymbol{adj}}\left(i,j\right)={\mathcal{F}}^{-1}\left({\boldsymbol{I}}_{\boldsymbol{M}}\left(i,j\right)\right)=\frac{1}{\sqrt{N^{\prime }-3}}\bullet \frac{e^{2{\boldsymbol{I}}_{\boldsymbol{M}}\left(i,j\right)}-1}{e^{2{\boldsymbol{I}}_{\boldsymbol{M}}\left(i,j\right)}+1}, $$

where *N* ^′^ denotes the assigned sample size and was set equal to *N*/4 in this study for the two groups were equally sized.

By the two MAGIC parameters, core *m*-modulated pairs were extracted and merged into a *m*-modulated gene interaction network. Here we defined three more modularity parameters to measure the size and information flow of the network, *Parameter 2: numbers of nodes* (genes), *Parameter 3: numbers of edges* (gene interactions), and *Parameter 4: connectivity* (average node degree). Statistical significance of the three parameters were tested by 10,000-time random permutations as described in Eq. 5.

### Functional annotation analysis and network visualization

Functional annotation analysis was performed by the Database for Annotation, Visualization and Integrated Discovery (DAVID) v6.7 [16, 17] to analyze the enrichment of key modulator genes in biological functions and processes. We focused on Gene Ontology (GO) terms of molecular functions, biological processes, and cellular components. We used the Functional Annotation Clustering tool to group GO terms to eliminate potential biases from highly similar terms. Gene interaction networks were analyzed and visualized by an open source software Cytoscape v3.2.1 [18], with nodes and edges representing genes and gene interactions, respectively, and node size denoting node degree.

## Results and Discussion

### Genome-wide identification of key modulator genes

The present study is aimed to systematically screen for key modulator genes from global gene expression data. As illustrated in Fig. 1, we selected and compared the samples with high (top 25%) and low (bottom 25%) expression of a candidate modulator gene *m*. Four parameters were designed to measure the modularity of *m* from two aspects, one at a genome-wide level and the others focusing on the core subnetwork only. ACI score (parameter 1) represents the overall change in interaction strengths between genome-wide gene interaction networks formed in the two sample groups. Focusing on the core sub-network (*m*-modulated gene interaction network) constructed merely by significantly changed edges, we further designed three parameters (namely, number of nodes, number of edges, and connectivity) to quantify the scale and information flow affected by the modulation of *m*. For each *m*, significance of the four parameters was tested by random permutation of dataset. Mathematical details are described in the Methods section.

### Properties of modularity parameters

**Fig. 2:**
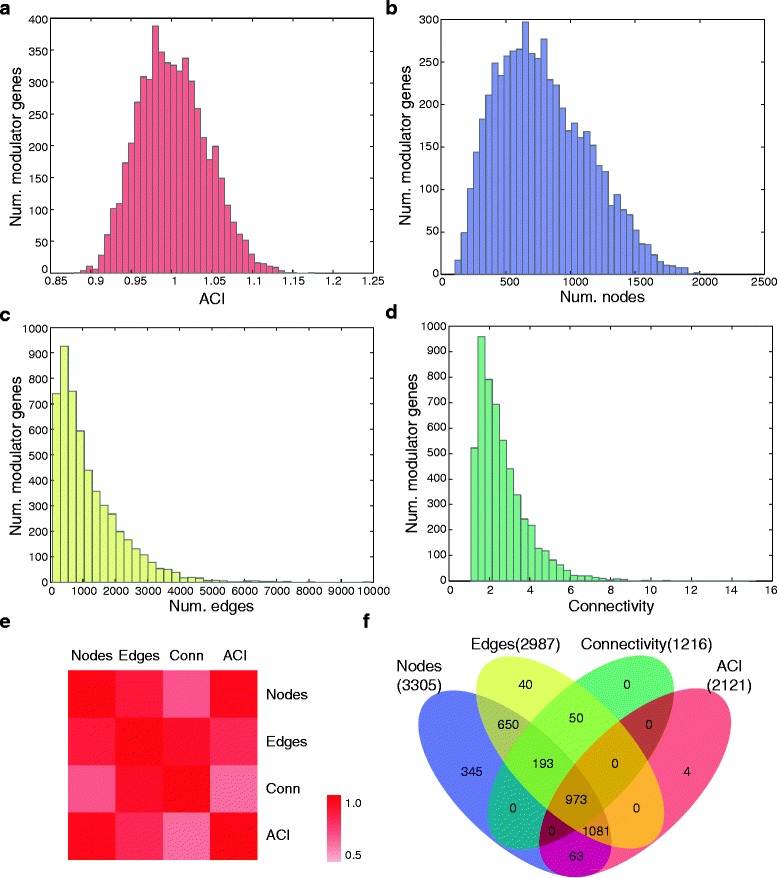
Identification of modulator genes in the discovery dataset. **a-d** Distributions of ACI score, number of nodes, number of edges, and connectivity of 5308 genes in the GSE2034 dataset. The parameters approximately followed log-normal distributions. **e** Pairwise correlation coefficients among the four parameters. Generally, the parameters were highly similar, with correlation coefficients falling between 0.64 and 0.95. **f** Venn diagram of significant modulators assessed by the parameters. Significance level of each parameter of a putative modulator gene was tested by a 10,000-time random permutation of the original expression dataset. By a cutoff of empirical *P*-value <0.0001, we identified 2121, 3305, 2987, and 1216 significant modulators by each parameter. A total of 973 genes reported by all of the parameters were defined as key modulator genes and selected for further analysis

Preprocessing of the discovery dataset, GSE2034, yielded expression profiles of 5308 unique and informative genes among 286 breast tumors. We analyzed each putative modulator by the modularity parameters. As shown in Fig. 2a-d, the parameters approximately followed log-normal distributions. At the genome-wide scale, the 5308 genes exhibited significantly intensified overall changes in interaction strengths than achieved by random permutations (mean ACI scores, 1.00 vs. 0.91; *t*-test *P*-value < precision of double-precision floating-point number, hereafter referred to as *P* ~ 0; Fig. 2a). Concordantly, each gene modulated a large core interaction network, with average numbers of nodes and edges as 789 (std., 355) and 1209 (std., 1033), respectively (Fig. 2b-c), compared to those formed by random permutations (mean, 152 and 98; *P*-values ~0). Substantial information flows underlie the modulated interaction networks (average connectivity of 5308 networks vs. randomness, 2.66 vs. 1.25, *P* ~ 0; Fig. 2d). Taken together, our data suggest that genes generally play roles as modulators to some extent, reinforcing the significance of modulation in gene interactions. We also investigated the similarity/distinctions among the four parameters. Pairwise correlation coefficients between these parameters ranged from 0.64 (connectivity vs. ACI score) to 0.95 (number of nodes vs. ACI score) (Fig. 2e), suggestive of the general agreements between the parameters.

### Identification and validation of key modulator genes

With the cutoff of empirical *P*-value at 0.0001, the four parameters reported 2121, 3305, 2987, and 1216 significant genes, respectively (Fig. 2f). We intersected these lists and identified a total of 973 key modulator genes for further analysis. We first examined ER, the best-studied modulator in breast cancer. Indeed, its encoding gene, *ESR1*, appeared to be the 258th modulator in the list, with individual ranks at top 512nd (9.6% of 5308 genes), 180th (3.4%), 193rd (3.6%), and 292nd (5.5%) with respect to each parameter. Only 86 genes (1.6%) outperformed *ESR1* by all parameters. At the top of the modulator list (Table 1), we identified a tumor suppressor gene in renal cell carcinoma, (*KANK1*) [19], a predictor of breast cancer progression and prognosis (*SFRP1*) [20, 21], and a marker gene of cisplatin sensitivity and tumorigenesis of cancers (*TMEM158*) [22, 23]. These novel modulator genes warrant further biological investigations.

**Table 1:** Top 20 modulator genes

Gene symbol	Num. nodes	Rank	Num. edges	Rank	Connectivity	Rank	ACI score	Rank
*KANK1*	1727	36	9909	1	11.48	4	1.15	2
*SFRP1*	1602	91	9772	2	12.20	3	1.10	62
*TMEM158*	1878	9	8132	3	8.66	11	1.14	4
*SLC16A1*	1891	5	7276	6	7.70	26	1.18	1
*POLD4*	1940	3	7198	7	7.42	31	1.14	7
*WWTR1*	1747	32	7180	8	8.22	18	1.13	9
*CYFIP2*	959	888	7278	5	15.18	1	1.04	643
*PPP1CB*	1844	16	6693	12	7.26	33	1.13	8
*FAIM3*	1342	384	7327	4	10.92	5	1.09	148
*ATP5G2*	1785	24	6218	20	6.97	40	1.14	3
*ITM2A*	1775	28	6612	14	7.45	30	1.12	24
*SYNM*	1557	120	6781	10	8.71	10	1.11	48
*GPM6B*	1664	58	6663	13	8.01	22	1.11	36
*IFRD1*	1809	20	6107	23	6.75	47	1.14	5
*LYN*	1844	15	6277	18	6.81	45	1.12	17
*CRYAB*	1514	159	6940	9	9.17	8	1.09	120
*GABRP*	1728	35	6184	22	7.16	37	1.13	13
*LY75*	1803	22	5988	25	6.64	51	1.13	12
*SERPINB5*	1638	69	6355	16	7.76	25	1.10	63
*UBE2E3*	1454	222	6355	17	8.74	9	1.10	85

Modulator genes are ranked according to average *z*-values of the four parameters

**Fig. 3:**
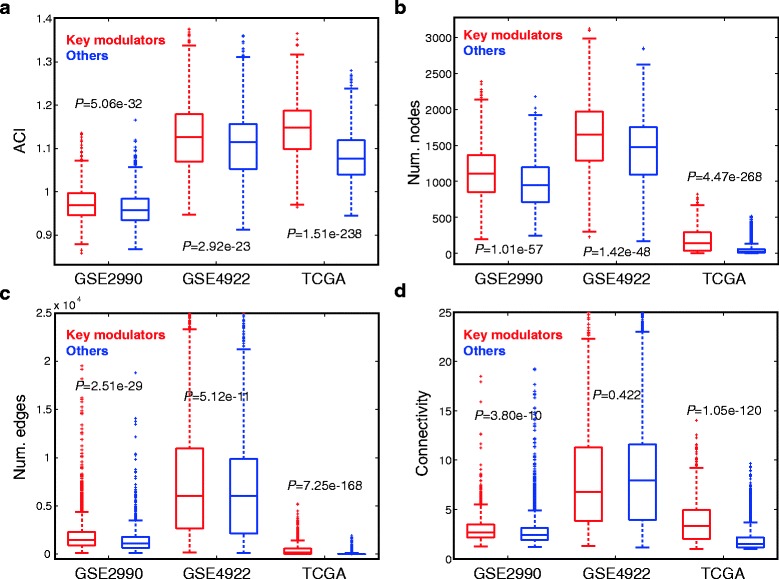
Validation of four modularity parameters in three independent cohorts. **a-d** Box plots comparing the ACI score, number of nodes, number of edges, and connectivity between 973 key modulators and other genes in three independent datasets. Statistical significance was assessed by the *t*-test. Generally, the key modulators exhibited significantly intensified modularity in the validation datasets, suggestive of the validity of the parameters and the conservativity of modulators among cohorts

Three independent datasets, GSE2990, GSE4922, and TCGA, of breast cancer were analyzed to verify the modularity of identified key modulators as well as the validity of the parameters. Notably, the 973 key modulators possessed significantly higher values of all parameters than other genes in all validation datasets (*t*-test *P*-values <3.8 × 10^−10^, except for connectivity in GSE4922, *P* = 0.42; Fig. 3). Overall, we corroborated the capability of the proposed workflow to identify known and novel modulators, validity of the parameters, and conservativity of key modulators among cohorts.

### *SFRP1*-modulated gene interaction network

**Fig. 4:**
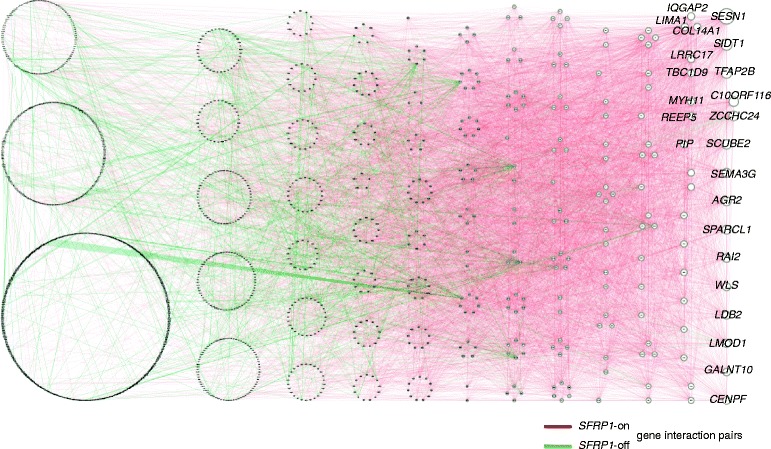
*SFRP1*-modulated gene interaction networks. We constructed the core interaction network modulated by a well-known prognostic gene, *SFRP1*, by merging the 9772 modulated interaction pairs among 1602 genes. With a connectivity of 12.2, the network was found quite intertwined. Gene pairs with significantly intensified correlation in *SFRP1*-on and -off samples are represented by red and green lines, respectively. Genes accounting for more than 1% of edges are labeled with gene symbols. Node size is proportional to degree

*SFRP1* was found the second-ranked modulator gene, with 1.10 ACI score, 1602 modulated nodes, 9772 edges, and 12.2 connectivity (Table 1). This secreted frizzled related protein is known to interact with and antagonize the Wnt signaling pathway [24, 25] and be a favorable prognostic factor in breast cancer [20, 21], prostate cancer [26], and glioblastoma [27]. Furthermore, it is dysregulated in tumor epithelium and tumor stroma [28] and altered in 12% of breast cancer (cBioPortal data [29, 30]). To investigated whether modulation accounts partly for the execution of its functions in breast cancer, we analyzed the core modulated gene interaction network and visualized it by the Cytoscape software. The 1602 modulated genes formed a highly intertwined network (connectivity, 12.2; Fig. 4), indicating the complexity of gene signaling mediated by *SFRP1*. Interestingly, the top 2 hub genes in the network, *SESN1* (degree, 220) and *SIDT1* (degree, 157) (Fig. 4), were reported to be involved in cell apoptosis and/or chemoresistance [31–33]. We also identified several hubs with uncharacterized functions in breast cancer, such as *TFAP2B*, *C10ORF116*, and *ZCCHC24*, that warrant further investigations.

We further studied the functions governed by *SFRP1* modulation with a DAVID analysis of genes involved in the network. Concordant to the prior knowledge of *SFRP1*, a significant association was found with the Wnt signaling pathway (Fisher’s exact test *P* = 0.0046). Functional Annotation Clustering of GO terms identified clusters of extracellular matrix (enrichment scores = 10.71, 6.56, and 5.77), response to hormone stimulus (score = 7.13), and cell cycle (score = 6.27) (Table 2), illuminating the involvement of *SFRP1* modulation in crucial functions in breast tumors and routine maintenance of cells.

**Table 2:** Top 6 clusters of GO terms enriched in *SFRP1*-modulated gene interaction network

GO ID	GO term	Num. genes	*P*-value
Cluster 1 (enrichment score: 10.71)			
GO:0005578	proteinaceous extracellular matrix	75	1.68E-13
GO:0031012	extracellular matrix	78	3.84E-13
GO:0044420	extracellular matrix part	37	7.39E-11
GO:0005201	extracellular matrix structural constituent	27	2.93E-08
Cluster 2 (enrichment score: 7.13)			
GO:0010033	response to organic substance	127	8.19E-12
GO:0009725	response to hormone stimulus	68	1.45E-07
GO:0048545	response to steroid hormone stimulus	42	6.50E-07
GO:0009719	response to endogenous stimulus	71	6.66E-07
GO:0043627	response to estrogen stimulus	27	4.33E-06
Cluster 3 (enrichment score: 6.56)			
GO:0044421	extracellular region part	157	2.80E-12
GO:0005615	extracellular space	99	1.56E-05
GO:0005576	extracellular region	232	4.66E-04
Cluster 4 (enrichment score: 6.27)			
GO:0007049	cell cycle	136	1.94E-12
GO:0022402	cell cycle process	104	6.36E-11
GO:0022403	cell cycle phase	78	7.27E-09
GO:0000279	M phase	61	6.77E-07
GO:0000278	mitotic cell cycle	66	9.25E-07
Cluster 5 (enrichment score: 5.80)			
GO:0000226	microtubule cytoskeleton organization	36	2.82E-07
GO:0007017	microtubule-based process	49	2.81E-06
GO:0007010	cytoskeleton organization	72	5.08E-06
Cluster 6 (enrichment score: 5.77)			
GO:0030198	extracellular matrix organization	32	5.00E-09
GO:0043062	extracellular structure organization	34	2.52E-05
GO:0030199	collagen fibril organization	12	4.00E-05

Clusters with more than five GO terms are represented by the most significant five

### Interactions and functions of key modulator genes

**Fig. 5:**
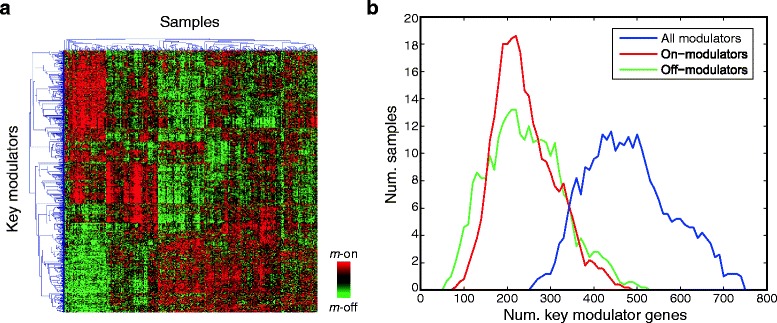
Interaction among key modulator genes. **a** Heatmap of expression profiles of 973 modulator genes in the discovery dataset. Samples and genes were hierarchically clustered with average linkage. Though the modularity parameters were generally correlated, distinctive clusters of samples and modulators indicate the substantial differences and functions underlying the 973 identified modulators. **b** Histograms of the numbers of on-, off-, and all modulators in a sample. In average, 241.5 on- (with top 25% expression among samples, red line) and off- (bottom 25%, green) modulators were found in each sample. Collectively, 483 key modulators (49.6% of 973, blue) functioned as “effective” modulators in a sample

We sought to analyze the interaction among the identified key modulator genes. While they all dominated a considerable scale of gene interactions, their expression profiles seemed to be non-identical. Unsupervised hierarchical clustering of expression data divided the modulator genes into three clusters and samples into five groups (Fig. 5a), implying the diversity of modulation patterns (a binary vector representing status of modulators of a sample [8]) across samples. Indeed, further analysis showed that each sample had on average 241.5 on- (with top 25% expression among samples) and 241.5 off- (bottom 25%) modulators among the 973 modulators (Fig. 5b). That is, roughly half (483 out of 973) of the key modulators were functioning as “effective” modulators in each sample; the maximum and minimum numbers of effective modulators per sample were 745 (76.6% of 973 modulators) and 280 (28.8%), respectively (Fig. 5b).

To investigate the landscape of biological functions governed by modulated gene interactions, we used the Functional Annotation Clustering tool of DAVID to identify enriched clusters of GO terms associated with the 973 key modulators. Interestingly, 4 of the top 6 clusters appeared to be immune/defense-related functions, including T cell activation (top cluster, enrichment score = 5.59; 52 modulators involved), defense response (2nd cluster, score = 5.29, 78 modulators), positive regulation of (alpha-beta) T cell activation/proliferation (4th cluster, score = 3.39, 63 modulators, and regulation of inflammatory response (5th cluster, score = 3.28, 37 modulators) (Table 3). Seven modulators were found common among these clusters: *CD24*, *CLEC7A*, *LYN*, *PTPRC*, *RIPK2*, *STAT5B*, and *TGFBR2*. Immunology and Immunotherapy are emerging fields in the prevention and treatment of cancers. In breast cancer, tumor-infiltrating lymphocytes (TILs) has the potential to serve as a predictive and prognostic biomarker and its variation is associated with patient subtypes (reviewed in [34, 35]). Furthermore, two early-phase clinical trials illuminated the promising responses of antibodies that target programmed cell death protein 1 (PD-1) and programmed death-ligand 1 (PD-L1) in the most adverse subtype of breast cancer, metastatic triple-negative breast tumors [36, 37]. Our data indicate that modulated gene interactions in part explain the significant effects of immune cells in breast cancer and warrant further investigations.

**Table 3:** Top 6 clusters of GO terms enriched in the 973 modulator genes

GO ID	GO term	Num. genes	*P*-value
Cluster 1 (enrichment score: 5.59)			
GO:0042110	T cell activation	26	5.08E-08
GO:0045321	leukocyte activation	38	5.64E-08
GO:0046649	lymphocyte activation	33	1.37E-07
GO:0001775	cell activation	41	2.14E-07
GO:0002521	leukocyte differentiation	23	5.96E-06
Cluster 2 (enrichment score: 5.29)			
GO:0006952	defense response	66	1.70E-06
GO:0006954	inflammatory response	42	2.16E-06
GO:0009611	response to wounding	55	3.64E-05
Cluster 3 (enrichment score: 3.60)			
GO:0007155	cell adhesion	66	1.03E-04
GO:0022610	biological adhesion	66	1.08E-04
GO:0016337	cell-cell adhesion	30	1.42E-03
Cluster 4 (enrichment score: 3.39)			
GO:0046635	positive regulation of alpha-beta T cell activation	11	9.27E-07
GO:0046634	regulation of alpha-beta T cell activation	12	3.69E-06
GO:0002684	positive regulation of immune system process	33	7.56E-06
GO:0051249	regulation of lymphocyte activation	24	1.39E-05
GO:0070665	positive regulation of leukocyte proliferation	14	1.41E-05
Cluster 5 (enrichment score: 3.28)			
GO:0050727	regulation of inflammatory response	15	1.04E-04
GO:0048584	positive regulation of response to stimulus	30	1.06E-04
GO:0050729	positive regulation of inflammatory response	9	2.31E-04
GO:0032101	regulation of response to external stimulus	21	8.89E-04
GO:0032103	positive regulation of response to external stimulus	12	1.03E-03
Cluster 6 (enrichment score: 3.07)			
GO:0010033	response to organic substance	68	7.95E-05
GO:0043627	response to estrogen stimulus	18	1.07E-04
GO:0048545	response to steroid hormone stimulus	26	1.24E-04
GO:0009725	response to hormone stimulus	36	2.60E-03
GO:0009719	response to endogenous stimulus	36	1.18E-02

Clusters with more than five GO terms are represented by the most significant five

Among the top GO clusters we also identified crucial tumor-related functions, such as cell adhesion (3rd cluster, score = 3.60, 66 modulators) and response to estrogen stimulus (6th cluster, score = 3.07, 68 modulators) (Table 3). The former is associated with metastasis and survival of breast cancer, while the latter is related to routine functions of hormonal receptors that were also seen in a previous co-modulation study [8]. Interestingly, in the cluster of response to estrogen stimulus, in addition to *ESR1* we identified another hormone receptor, androgen receptor (*AR*). Taken together with the well-studied role of ER as a modulator gene in breast cancer, our data showed that its functions, especially in the response to estrogen, are co-performed by other modulator genes, highlighting the essential involvement of modulation in such functions.

### Limitations and future work

We measured the modularity of each putative modulator at two layers of interaction networks, one focusing on global changes and the other on a core subset of network. Four modularity parameters were designed accordingly, of which validity was confirmed by three independent datasets. However, for the nature of gene modulation as an indirect and complex mechanism, there may exist other parameters that could better measure modularity when cooperatively considered with the proposed four parameters. Furthermore, since the statistical features of the four parameters have not been characterized, we employed random permutations to assess the statistical significance, which limits the computation efficiency and statistical stringency. Out of simplicity, we compared the interaction networks formed in *m*-on and -off samples. However, modulated gene pairs of which correlation changes gradually with the continuous-state expression of *m* [38] may be omitted. Besides, in the study we assumed modulation effects to be independent events. Though, biological intuition is that several modulators may jointly modulate a common pair of genes [8], and pairs of genes modulated by a modulator may have competing effects against each other [39]. Future investigation addressing the limitations may further unveil a comprehensive map of modulated gene interactions in cancers and other diseases.

## Conclusions

This study addresses the need for a genome-wide screening for key modulator genes of gene interaction. We developed a systematic workflow that incorporates a correlation-based modulation analysis of gene interaction networks. About one thousand key modulators were identified, including the best-known modulator *ESR1* and other novel ones, and validated in independent cohorts. These modulators were associated with hormone signaling and immune/defense-related and tumor-associated functions. Overall, this study is, to our knowledge, the first to screen for and investigate modulator genes in breast cancer on a genome-wide scale. The proposed workflow is widely applicable to other cancers and expected to unveil the landscape of modulated gene interactions.
